# Profile of Irish female GPs and factors affecting long-term commitment: a descriptive study

**DOI:** 10.3399/BJGPO.2023.0229

**Published:** 2024-08-21

**Authors:** Ivana Keenan, Laura Cullen, Gabrielle Hogan, Noirin O’Herlihy, Ciara McCarthy, Claire Collins

**Affiliations:** 1 Irish College of General Practitioners, Dublin, Ireland; 2 Marino Medical Centre, Primary Care Centre, Bantry, Ireland; 3 University College Cork, Cork, Ireland; 4 Red House Family Practice, MPHC, Mallow, Ireland

**Keywords:** female, general practice, general practitioners, Ireland, primary health care, workforce

## Abstract

**Background:**

Over the past two decades, many countries have reported an increased percentage of female staff in the general practice workforce. Considering the importance of general practice workforce planning, it is necessary to investigate the current working patterns of female GPs.

**Aim:**

To describe the female GP workforce in Ireland and to investigate factors that may affect their long-term commitment to general practice.

**Design & setting:**

Descriptive, cross-sectional study conducted with female GPs in Ireland.

**Method:**

A ‘membership survey’ was emailed to 1985 female GPs in November 2021. In total, 345 female GPs responded, providing a response rate of 17.4%.

**Results:**

The study revealed that a majority of the female GP workforce in Ireland worked in the position of the GP principal (62.3%), but also provided out-of-hours services (64.3%), and undertook caring responsibilities (84.1%). In total, 41.2% of the responders disclosed having at least one paid additional role, mainly in the field of academia and teaching. Most female GPs worked fewer than eight clinical sessions a week (80.5%). GPs who held General Medical Services (GMS) contracts (72.8%) were significantly more likely to work more clinical sessions a week and had been longer employed in general practices (>5 years) than GPs who did not have GMS contracts.

**Conclusion:**

Irish female GPs demonstrated a significant adjustment of their working patterns, including reducing their number of clinical sessions and balancing between additional roles, to ensure their long-term commitment to general practice. Current practices and vision on GP roles must be recognised and supported to allow for adequate workforce planning.

## How this fits in

The general practice workforce is undergoing a significant change across the globe. Due to workforce and workload challenges, increasing numbers of GPs are changing their working life and career path by reducing the number of clinical working sessions, developing portfolio careers, and/or considering emigration. As women constitute a high proportion of the general practice workforce, this study provides valuable insights into their working patterns; for example, most female GPs work as GP principals, concurrently undertaking various caring duties and additional non-clinical roles. This study describes the situation of female GPs in Ireland; however, the lessons apply to all healthcare systems experiencing similar trends.

## Introduction

General practice is recognised as a cornerstone of primary health care and is an essential part of any functional health system.^1^ For patients in many countries, GPs represent the first point of contact, and play a central role in the prevention, early detection, and treatment of a wide range of morbidities.^2–4^ However, a shortage of GPs across European countries has been reported,^4–8^ causing significant challenges in providing timely and high quality patient care. Ireland is not an exception. Several reports highlighted an increased demand for the GP workforce in Ireland,^9–11^ outlining that the provision of a sufficient response to patients’ demands requires an increase of 42% in the number of GPs.^11^

Furthermore, in the past decade, general practice has undergone significant change, including the increased participation of females in the profession.^12–15^ In many countries the majority of GPs are female, with women accounting for 51% of the GP workforce in Australia,^16^ followed by 53% in the UK,^17^ 56% in Ireland,^18^ and more than two-thirds in Latvia, Estonia, Lithuania, and Romania.^19^ Ireland, like other countries, is seeing an increased feminisation of general practice, with higher proportions of females among younger cohorts and trainees.^4^

Previous studies have highlighted the growing number of female GPs committing to work fewer clinical sessions than their male colleagues.^20–23^ Working patterns of female GPs are often determined by additional roles undertaken, either through the development of portfolio careers and/or diverse caring roles.^23–25^ Research from the United States and Canada has highlighted that female GPs spend significantly more time in childcare duties than their male colleagues.^26,27^ Female GPs frequently undertake unpaid caring roles in their personal lives, and find it harder to remain in the full-time workforce.^27^ A systematic review published in 2020 stressed that competing demands of being a mother and a doctor could be balanced by supportive maternity leave and return-to-work policies.^28^ A lack of supportive policies causes higher turnover among female GPs, whereby GP mothers of young children are less likely to intend to leave but often do so, suggesting that their leave is unplanned.^29^ This contributes to workforce shortages that are more difficult to predict.

Work roles and demands of the job are often closely related to the obligations, entitlements, and payments proposed in employment contracts for GPs. While GP employment contracts differ across Europe,^30^ access and requirements to them can impact career choices.^31^ In Ireland, GPs generally operate under a dual public payment and private payment system.^30^ The public aspect is capitation-based and individual GPs hold a contract with the Health Service Executive to provide specific healthcare services to those patients eligible for same. Obtaining this contract, known as a General Medical Services (GMS) contract, is considered a major milestone in the career progression of Irish GPs.^32^ The contract is a fundamental source of income for the majority of GP practices nationwide. Given the importance it has to the financial survival of GP practices, it often marks the point of a GP making a long-term commitment to practising in a particular area. However, the requirements for entry into the scheme may be a factor in why some GPs chose not to proceed with it or are not eligible.^32,33^

Changes in doctors’ working patterns are a challenge to planning the long-term provision of effective health services. Considering that the majority of GPs are female, characterisation of the female GP workforce and examination of their career choices is essential for planning a sustainable future medical workforce. The aim of this article is to describe the female GP workforce in Ireland and to investigate factors that may impact their long-term commitment to general practice, including clinical sessions, personal commitments, and perception of contract requirements.

## Method

The Irish College of General Practitioners (ICGP), being Ireland’s professional body for education, training, and standards in general practice, undertakes an annual ‘membership survey’ to consult its members. The membership survey assessed for the article was sent in November 2021, and all 3732 ICGP members were invited to participate. The data from the female responders are the focus of this article. The survey was developed and distributed using the online platform SurveyMonkey. The initial survey was sent by email, with a follow-up reminder issued to all non-responders. Only consented participants were able to respond to the questions.

The survey contained four main sections: the participant’s demographic profile; current employment status and structure of the practice participants worked in; attitudes towards the GMS contract; and assessment of the work dynamic, including experiences of physical or verbal abuse by patients and stress in the workplace. The employment status was determined by applying the guidelines from ‘A Handbook for the Establishing General Practitioner’.^34^ This document gives the following definitions of positions within general practice:

- GP principal: main stakeholder in the practice, holding financial, property, and employment responsibilities;
- GP assistant: has a strong commitment to practice and takes on clinical responsibilities;
- Regular sessional GP: a part-time employee whose work generally consists of a combination of regular sessions in one or more practices;
- Salaried partner: also a principal, who shares profits and responsibilities in an agreed proportion with the other practice partner;
- Locum GP: a GP who temporarily manages issues until the regular GP returns.^34^

The participants working hours were assessed as the number of sessions per week, where the session is defined as half a working day (1–4 sessions = ≤2 working days, 5–6 sessions = 3 working days, 7–8 sessions = 4 working days, ≥9 sessions = 5 working days).

The survey required approximately 15 minutes to complete. The majority of the responses were analysed through single-choice and multiple-choice questions by means of descriptive statistics (count, mean, percentages). Perceptions of stress were collected through a Likert 5-point scale, where 0 indicated no stress, and 5 indicated very stressed all the time. Pearson’s χ^2^ test, Welch two-sample *t*-test, and Spearman correlations were used to determine whether a statistically significant relationship existed between observed and expected values (*P* values <0.05 were considered statistically significant). The analysis was carried out using SPSS Statistics software (version 27).

The participants were also given the opportunity to expand on some of their responses within free-text sections throughout the survey. The free-form text data were analysed by applying qualitative content analysis.

## Results

The survey was sent to all members of the ICGP: 3732 GPs who are employed in Ireland. Of this number, 1985 (*n* = 53.2%) were female GPs. Within the female cohort, 345 female GPs responded to the survey, providing a response rate of 17.4%. This article is focused only on the female cohort.

### Profile

Of the 345 female survey participants, 58.9% (*n* = 203) were aged under 50 years, and 42.9% (*n* = 148) had worked less than 15 years in general practice. The majority of female GPs (62.3%) worked in a GP principal position, which requires managerial skills and further practice responsibilities, followed by a full-time or part-time assistant role (17.5%). Most female responders worked in group practices with two to three doctors (42.5%, *n* = 142) (Table 1).

**Table 1.** Profile of female GPs

	%	*n*
**Age, years (*n* = 345)**		
<40	21.5	74
40–49	37.4	129
50–59	27.5	95
60–64	10.7	37
≥65	2.9	10
**Years worked in general practice (*n* = 345)**		
<5	10.4	36
5–14	32.5	112
15–29	40.0	138
>29	17.1	59
**Current working position in general practice (*n* = 342)**		
GP principal	62.3	213
Full-time or part-time assistant	17.5	60
Regular sessional GP	7.3	25
Salaried partner	5.3	18
Locum GP	1.8	6
Other (please specify)	5.8	20
**Practice size (*n* = 334)**		
Single-handed	12.3	41
Group practice with 2–3 doctors working full-time or part-time	42.5	142
Group practice with 4–6 doctors working full-time or part-time	32.6	109
Group practice with >6 doctors working full-time or part-time	10.2	34
A practice managed by a corporate entity that manages a number of practices	2.4	8
**Practice location (*n* = 342)**		
City (population ≥50 000)	41.8	143
Town (population 1500–49 999)	45.0	154
Village (population <1499)	13.2	45

### Additional roles

Besides their main role as a GP, many participants also had additional responsibilities, including the provision of out-of-hours (OOH) services, additional paid positions, and caring responsibilities. According to the survey, most female GPs (64.3%, *n* = 222) provided OOH services. While OOH commitment per month may vary, GPs reported working an average of 7.5 hours a month in this role.

The survey also revealed that 41.2% (*n* = 142) of the responders disclosed having at least one paid additional role. Within that cohort, 75.4% (*n* = 107) disclosed having one more additional paid role to their GP position, and 24.6% (*n* = 35) had two or more paid roles in addition to their GP position. The additional paid positions varied greatly, including roles in ‘undergraduate/postgraduate teaching’ (*n* = 65), ‘academic’ (*n* = 24), ‘hospital medicine’ (*n* = 21), and ‘other’. By analysing additional paid roles and the number of clinical sessions a week, we found a correlation indicating that GPs who have at least one additional paid role were more likely to work fewer clinical sessions a week in general practice than GPs who did not have additional paid roles (*P*<0.001) (Table S1).

**Figure 1.**
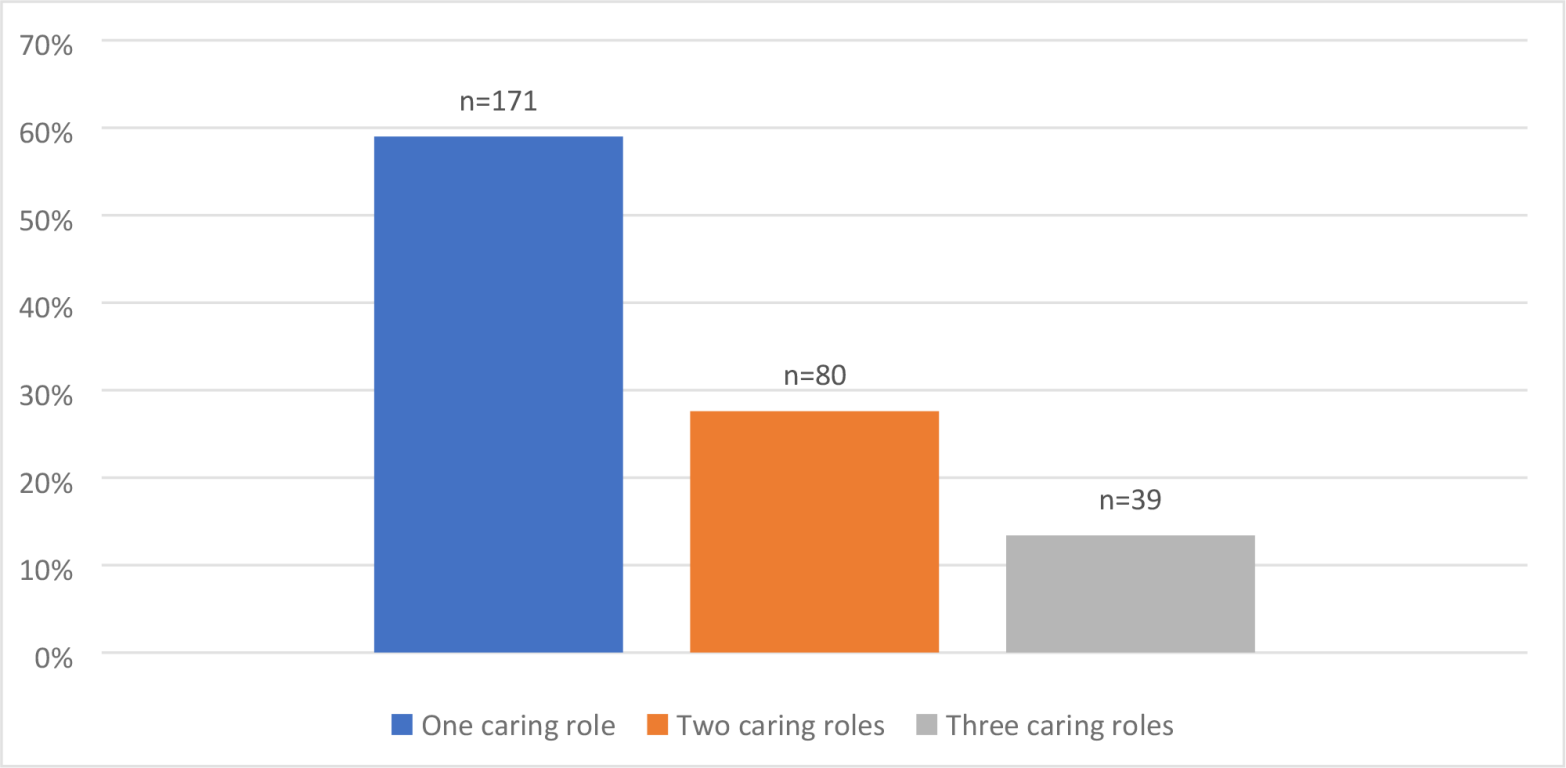
Division of caring roles undertaken by female GPs (*n* = 290)

The vast majority of responders (84.1%, *n* = 290) also disclosed having at least one caring responsibility, including caring for children, older relatives, and/or partners. As per Figure 1, some female GPs had two or three caring responsibilities in addition to their employment commitments. By analysing the statistics on female GPs with at least one caring role and the number of clinical sessions a week, we found a correlation indicating that GPs who have at least one caring role were less likely to work 5 days a week in general practice than GPs who did not have an additional caring role (*P*<0.001) (Table S1).

### Current working sessions

**Figure 2.**
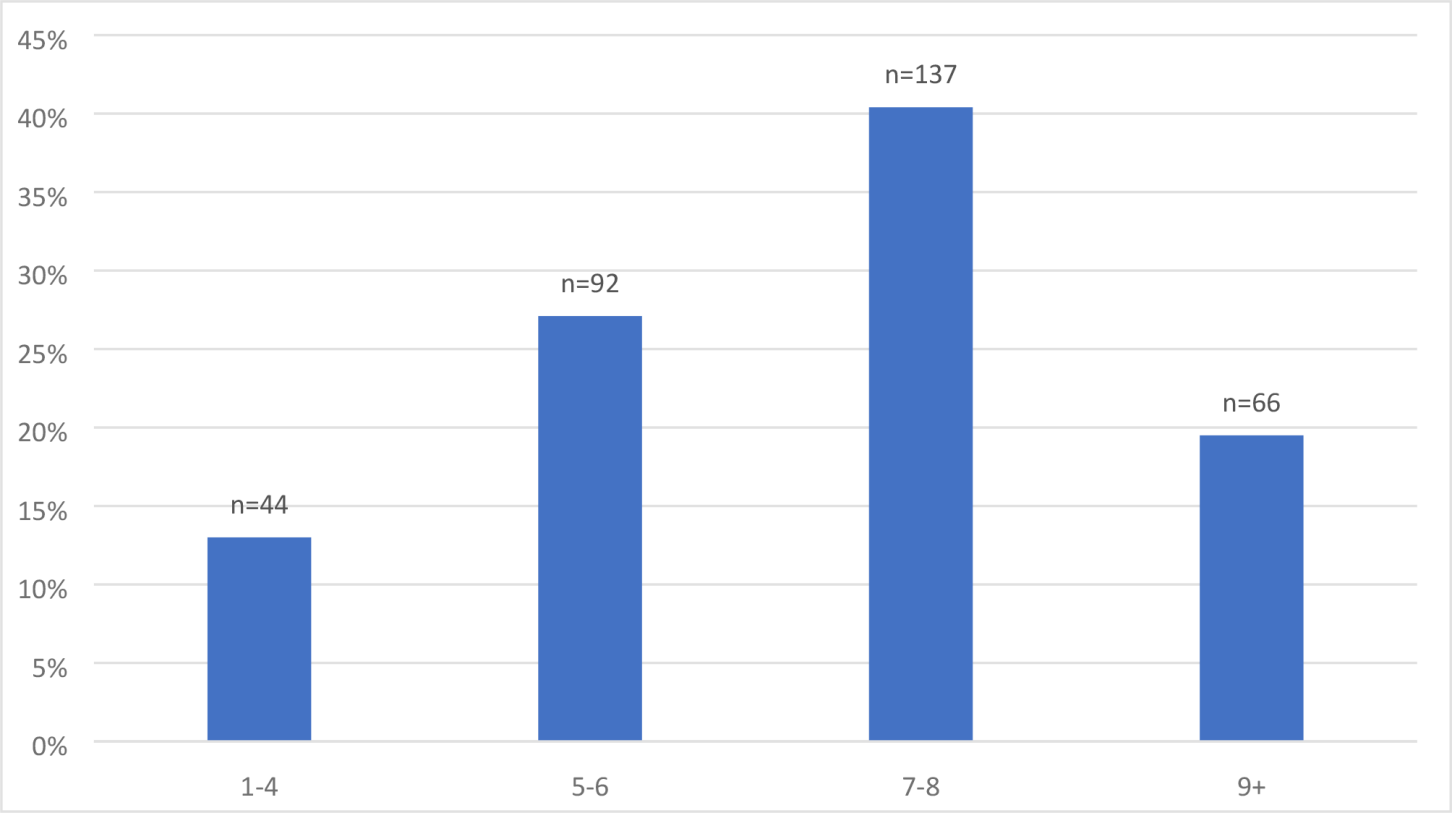
Number of working sessions a week by female GPs (*n* = 339)

The vast majority of the female GPs reported working eight or fewer clinical sessions a week (80.5%, *n* = 273), whereas fewer than 20% of participants disclosed working 5 days a week in general practice (*n* = 66) (Figure 2).

Welch two-sample *t*-test showed that GPs who worked nine or more clinical sessions a week were more likely to hold GMS contracts (*P*<0.001) and be in single-handed practices (*P*<0.001) than GPs who worked fewer than nine sessions a week (Table S1).

### GMS contract

**Figure 3.**
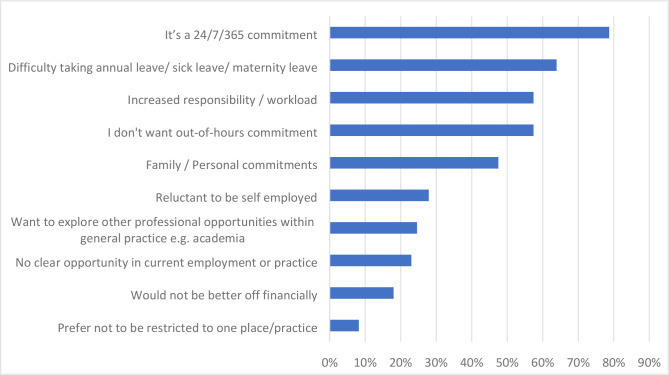
Female GPs' reasons for not holding the GMS contract (*n* = 61)

In total, 72.8% (*n* = 251) of female GPs held the GMS contract. Responders who did not have the GMS contract (27.0%, *n* = 93) were also asked if they were planning to take up a GMS contract in the future; out of the 91 respondents 42.9% (*n* = 39) said ‘No’, 24.2% (*n* = 22) were ‘Not sure’, and 33.0% (*n* = 30) said ‘Yes’. The most cited reasons for lack of interest in holding the GMS contract were ‘It’s a 24/7/365 commitment’ (78.7%, *n* = 48) and ‘Difficulty taking annual leave/sick leave/maternity leave’ (63.9%, *n* = 39) (Figure 3).

By analysing years working in general practice and GMS contract-holding status, we found a correlation indicating that female GPs who worked longer in general practice were more likely to hold GMS contracts (*P*<0.001) (Table S2).

### Work dynamic: experiences of abuse and stress in the workplace

In total, 300 (86.9%) participants disclosed being subjected to abuse by patients (physical or verbal) in the course of their work as a GP. Of this number, 72.0% (*n* = 216) experienced abuse in the past year, and 40.7% (*n* = 122) more than a year ago; noting that some responders reported experiencing abuse in both periods.

The survey also asked female GPs how well they were coping with various personal and professional challenges currently. On a scale of 1–5, the average level of stress experienced was found to be 3.60. On average, stress levels were higher among GPs who were employed as principals, who worked for ≥9 sessions a week, and who experienced abuse (Table 2).

**Table 2.** Average levels of stress among female GPs

Work characteristics	Mean stress levels	Test run	*P* value	Significance
**Sessions per week**		Welch two-sample *t*-test	0.018	*
≤8 sessions a week	3.53			
≥9 sessions a week	3.86			
**Practice size**		Welch two-sample *t*-test	0.783	na
Single-handed practice	3.66			
Group practice	3.61			
**Caring duties**		Welch two-sample *t*-test	0.152	na
Yes	3.62			
No	3.61			
**Additional paid roles**		Welch two-sample *t*-test	0.109	na
Yes	3.68			
No	3.48			
**GMS contract**		Welch two-sample *t*-test	0.103	na
Yes	3.70			
No	3.31			
**Position in the practice**		Welch two-sample *t*-test	<0.001	***
GP principal	3.78			
Other	3.29			
**Experience of abuse**		Welch two-sample *t*-test	0.015	*
Yes	3.66			
No	3.14			

GMS = General Medical Services.

## Discussion

### Summary

The study revealed that a majority of the female GP workforce in Ireland work in the position of the GP principal (62.3%), but also provide OOH services (64.3%), and undertake additional caring responsibilities (84.1%). In total 41.2% of the responders disclosed having at least one paid additional role, mainly in the field of academia and teaching. Female GPs are likely to work fewer than eight clinical sessions (80.5%), which is often considered less than full-time employment. GPs who worked fewer than four clinical sessions are more likely to have additional paid roles, work in group practices, and were less likely to undertake GMS contracts than GPs who worked more clinical sessions. Previous studies echoed that while many GPs work less than full-time clinical hours, they are often enrolled in full-time paid employment across a range of alternative roles outside general practice.^20,35^

As the GMS contract requires 40 hours a week of health care for public patients,^32^ usually arranged between GPs employed in the practice, this type of commitment is often less appealing to GPs employed outside of clinical general practice. The main reasons for not intending to take up a GMS contract in the future were the contract being perceived as a ‘24/7/365 commitment’, and due to ‘Difficulty taking annual leave/sick leave/maternity leave’. Of note, the male counterparts in the survey reiterated the views of their female colleagues, reporting the same reasons. Regarding the workplace environment, among female responders who experienced abuse, the majority (72.0%) reported experiencing physical or verbal abuse by their patients in the last year; this was higher than the male cohort from the same survey (Table S3 provides a comparison between the female and male responders in our study across all variables). The mean level of stress was 3.60, with slightly higher rates when employed ≥9 sessions a week or in the position of GP principal.

### Strengths and limitations

The current study provides a unique perspective on the female GP workforce in Ireland. A variety of survey questions allowed investigation of responders’ profiles and captured data on their current working commitments, allowing for future workforce planning.

Although a low response rate is in line with previous ICGP membership surveys, a limited study sample decreased the representativeness of the study population. Time constraints are often identified as the main barriers to survey participation among healthcare professionals.^36^ Considering the value of GP perspectives on workforce planning, the development of strategies for increasing GP involvement in research activities is important.

As the survey was voluntary, self-selection bias may be present; there is therefore a possibility that the sample is not representative of the wider population of female GPs in Ireland, which may result in underestimates or overestimates.

Furthermore, ‘sessions worked’ is considered a crude measure of workload, and therefore does not adequately assess the number of hours worked, the complexity of the work, or the administrative load associated with those sessions. The membership survey asked questions in such a way that it is not possible to elucidate this, but consideration will be given to wording for future research in the area to assess workload in more detail.

Finally, the results of this study do not necessarily reflect all female GPs in Ireland. Firstly, the cohort of this study is women who were currently employed as a GP, the viewpoints of women who decided to leave general practice are not represented. Also, an additional limitation to consider is the possibility that those female GPs who worked fewer hours were more likely to respond to the survey and therefore may have been overrepresented, which limits the generalisability of the findings to the broader cohort.

### Comparison with existing literature

The findings of our study were in accordance with the previous research, which highlighted that many female GPs decide to work part-time or fewer than eight clinical sessions a week.^20–22^ a necessity for more flexible and part-time employment is often due to additional roles and unpaid caring responsibilities that female GPs undertake.^26,27,35^ reducing the number of clinical sessions is often pursued to achieve a balance between professional and personal life. However, this employment approach may pose challenges, and it often entails more time pressure and lower pay.^21^ lower remuneration can result in a loss of control over the type of work performed, potentially worsening work–life conflict, which is associated with turnover intentions and burnout.^21^

Furthermore, the literature on female GPs highlighted that additional roles and responsibilities cause changing aspirations to a long-term commitment to general practice. The present study found that 84.1% of female GPs have at least one caring responsibility. Caring responsibilities are often seen as major barriers to women’s participation in general practice, and therefore reduced clinical sessions and adoption of portfolio careers became highly desirable to attain a healthy balance between work and personal life.^24,37^ The rise of portfolio careers was especially evident in the last decade when this career pathway was chosen as it provides career diversity and flexibility, as well as adaptability to desired lifestyles.^38,39^ GPs with portfolio occupations hold additional specialist medical roles or are actively engaged in academia, education, and research,^24,25^ which was in line with our study findings. Although portfolio careers are seen as greatly beneficial for medical practitioners,^24,25,29^ issues of fragmented care and loss of interest in continuing working primarily as a GP are raised as disadvantages that erode the GP’s traditional role.^24,25,29^

### Implications for research and practice

Overall, the GP workforce in Ireland and elsewhere is changing, with female GPs working fewer clinical sessions and choosing additional employment roles, while undertaking multiple caring responsibilities. Policymakers and the GP profession must recognise and respond to the evolving expectations and needs of female GPs, who now make up the majority of the GP workforce, and be open to and support new ways of working. Although some changes have already taken place regarding an increased number of multi-doctor practices, these changes are largely reactionary, and there is further scope for more proactive work to be done at a central, political level. Additional resources and improved retention strategies are needed to support the career expectations of female GPs and ensure strong female representation in future healthcare planning.

Both Irish society and the profession of general practice in Ireland have changed irrevocably since the inception of the GMS contract in 1972 and its revision for financial purposes in 1989. Our research shows that while women now comprise the majority of GPs, some are not opting to sign the GMS contract because of the perceived demands it places on them. Therefore, a close examination of the structural frameworks that the general practice profession is built on, such as the GMS contract, is necessary, as outdated employment contracts might act as a barrier at an institutional level to the full participation of a significant proportion of GPs.

Considering that the recruitment and retention crisis has been taking place right across all the professions of medicine, including general practice, a clear understanding of not only how male and female GPs in Ireland work, but also the motivations around work, is needed. Future studies should apply a qualitative research lens to allow for an in-depth exploration of GPs’ experiences and expectations of a sustainable career in general practice. We need to accept responsibility for the norms that exist in the profession of general practice and take steps to address practice-level and system-level organisational factors that must be changed to bolster and appreciate all the roles that GPs play.

## Data

The dataset relied on in this article is available from the corresponding author upon reasonable request.
